# Component analysis of a self‐monitoring intervention for increasing task engagement for individuals with developmental disabilities

**DOI:** 10.1002/jaba.70053

**Published:** 2026-02-06

**Authors:** Erin Leif, Eileen Roscoe, Lauren Rae, Sam Sheets

**Affiliations:** ^1^ Faculty of Education Monash University Melbourne Victoria Australia; ^2^ Department of Psychology Western New England University, Springfield, Massachusetts, USA and the New England Center for Children Southborough Massachusetts USA

**Keywords:** differential reinforcement of alternative behavior, engagement, self‐management, self‐monitoring

## Abstract

Self‐monitoring (SM) has been used as part of intervention packages to enhance skills such as leisure and vocational engagement for individuals with intellectual and developmental disabilities (IDD). However, the effectiveness of SM alone remains unclear. We analyzed components of an SM intervention to increase task engagement for five individuals with IDD. Participants were first taught to accurately self‐monitor their engagement. Sequential analyses evaluated SM alone, SM + differential reinforcement (DR) for accurate SM, SM + DR for accurate SM and task engagement, and DR for task engagement. SM alone was ineffective. Combining SM with DR for accurate SM improved accuracy of SM for all participants but increased task engagement for only two. Combining SM with DR for both accurate SM and task engagement increased engagement for the remaining participants. High levels of task engagement were maintained when reinforcement for engagement was provided without SM. Implications for intervention design are discussed.

Individuals with intellectual and developmental disabilities (IDD) can acquire various skills that increase their independence such as academic task completion (Alresheed et al., 2018), vocational skills (Campanaro et al., 2021), and leisure activity engagement (Leif et al., 2020). However, independent performance of these skills often does not maintain, requiring continued caregiver support (e.g., via prompting and programmed consequences; Hume et al., 2009). As a result, individuals with IDD may experience fewer opportunities to participate in leisure and recreational activities (Haythorne et al., 2022) or employment (Bross et al., 2021) and may experience continued placement in more restrictive educational or living environments (Kurth et al., 2014; Phillips & Rose, 2010). For this reason, it is important to identify interventions for promoting maintenance of skills.

One potential intervention component for promoting maintenance of skills for people with IDD is self‐monitoring (SM). SM is defined as the process of observing and recording one's own behavior (Cooper et al., 2020) and typically involves providing access to materials for recording one's own behavior. Examples of these materials include paper with boxes to check (e.g., Koegel & Koegel, 1990), tokens (e.g., Newman et al., 1997), tally counters (e.g., Koegel et al., 1992), or digital applications on mobile devices (Cook & Sayeski, 2022). These SM materials may function as discriminative stimuli that signal the availability of reinforcement for task completion (Hume et al., 2009). Several studies have demonstrated the effectiveness of SM for increasing appropriate behavior for individuals with IDD such as academic task engagement and completion (e.g., Kumm et al., 2021; Risse et al., 2023; Rosenbloom et al., 2019), vocational engagement and productivity (e.g., Ackerman & Shapiro, 1984), leisure item engagement (e.g., Stahmer & Schreibman, 1992), appropriate social interactions (e.g., Koegel et al., 1992; Parker & Kamps, 2011; Sainato et al., 1992), and activities of daily living (e.g., Bouck et al., 2014; Pierce & Schreibman, 1994).

One potential explanation for prior positive research findings related to SM promoting appropriate behavior is its frequent inclusion as a component within multicomponent interventions that also include prompts or reinforcement for appropriate behavior (Bruhn et al., 2022; Howard et al., 2020). Bruhn et al. (2022) conducted a systematic review of single‐case research studies on SM in school settings, and the strongest effects on academic engagement were found in studies combining SM with differential reinforcement (DR) for engagement. Other studies have found similar effects. For example, Parker and Kamps (2011) implemented a multicomponent SM intervention that included presenting an SM data sheet, vocal and physical prompts to engage in the task, and reinforcement of task completion to teach two participants with autism to complete leisure activities (playing games, cooking, and going to a restaurant). Contingent on completion of all task analysis steps and inserting checks in all boxes on the SM data sheet, the experimenter presented participants with a preferred activity. The multicomponent SM intervention increased task completion for both participants. Risse et al. (2023) extended Parker and Kamps by providing reinforcement contingent on accurate SM as part of an intervention for increasing academic task completion displayed by three students with attention‐deficit/hyperactivity disorder and specific learning disabilities.

Parker and Kamps (2011) and Risse et al. (2023) demonstrated that interventions that include SM can increase the appropriate behavior of individuals with autism, attention‐deficit/hyperactivity disorder, and learning disabilities. Unfortunately, across these studies, the independent contribution of the SM component was unclear because multiple intervention components were conducted concurrently with the introduction of SM materials. One way to evaluate the independent contribution of the SM component is through a component analysis. For instance, Storey and Gaylord‐Ross (1987) evaluated the effects of SM alone and in combination with DR for increasing social interaction (i.e., providing praise to another individual) for individuals with IDD. SM alone and in combination with DR were both effective for increasing social interaction. However, data on accurate SM during SM instruction and intervention were not provided. Additionally, the SM alone condition was always preceded by the SM + DR condition. Therefore, the extent to which participants' SM was accurate and whether an immediate prior history of DR was required for effectiveness was unclear.

In the studies above, information was not provided about whether and how participants were trained to self‐monitor their behavior and no data were reported on the accuracy of participants' SM. To address these limitations, Fritz et al. (2012) conducted a component analysis of SM for decreasing stereotypy with three participants with IDD. Before the treatment component analysis, participants were taught to record whether stereotypy occurred or did not occur while a confederate engaged in the participant's topography of stereotypic behavior, by placing a mark in the corresponding box on a data sheet when a tone sounded. Accurate recording resulted in a preferred edible, and inaccurate responses resulted in a correction procedure (erasing the inaccurate mark and prompting the participants to mark the correct box). After participants demonstrated mastery of accurate recording of the confederate's stereotypy, the SM + DR (accurate) phase was initiated, in which praise and a preferred edible were delivered for accurate SM at the end of each interval. Two additional intervention conditions were conducted: SM + DR (accurate) with differential reinforcement of other behavior (DRO) and DRO alone. The SM + DR (accurate) with DRO condition was similar to the SM + DR (accurate) condition, except that the experimenter delivered praise and an edible only if the participant both refrained from stereotypy for the entire interval and accurately recorded the absence of stereotypy when the timer sounded. During the DRO‐alone condition, the SM materials were removed and the experimenter delivered praise and an edible if the participant refrained from stereotypy for the entire interval. Different outcomes were obtained for each participant. For one participant, SM + DR (accurate) was effective, and the authors determined that this was because the SM response functioned as a competing response (i.e., a similar academic task, copying words, was found to be similarly effective). For another participant, SM + DR (accurate) was initially ineffective but subsequently was effective if it followed the SM + DR (accurate) with DRO condition. For the third participant, neither SM + DR (accurate) nor SM + DR (accurate) with DRO were effective; however, DRO alone was effective for decreasing stereotypy. Fritz et al. hypothesized that SM + DR (accurate) was ineffective but that DRO was effective because this participant did not consistently exhibit accurate SM. As a result, SM + DR (accurate) may have been associated with a lower reinforcement rate than DRO alone.

The purpose of this study was to extend previous research on SM by conducting a component analysis to isolate and evaluate the independent and combined effects of SM and DR on task engagement, productivity, and accurate SM for individuals with IDD. This study extended prior work by Fritz et al. (2012) in using SM to increase task engagement rather than reduce stereotypy. We extended their procedures in four key ways: (a) participants were taught to self‐monitor their own behavior (rather than a confederate's), (b) the effects of SM materials alone (i.e., without reinforcement or prompting) were evaluated after SM instruction to examine whether SM alone could increase task engagement and accurate SM, (c) the additive effects of DR for accurate SM and for task engagement were examined to determine which components were necessary and sufficient for increasing task engagement and accurate SM, and (d) a treatment preference assessment was conducted with a subset of participants to explore their relative preference for SM‐based versus reinforcement‐only conditions.

Accordingly, the study addressed four research questions: (1) Does task engagement and accurate SM maintain following SM instruction when SM materials are provided in the absence of reinforcement? (2) Does SM + DR (accurate) or SM + DR (accurate + engagement) increase task engagement and accurate SM? (3) Does SM enhance the effects of DR (alone) for task engagement? and (4) Do participants show a preference for intervention conditions that include SM? To answer these questions, we used a treatment component analysis embedded within a reversal design and evaluated behavior across four conditions: SM (pre‐ and postinstruction), SM + DR (accurate), SM + DR (engagement and accurate), and DR (engagement only). Following the treatment component analysis, a preference assessment was conducted using a concurrent chain schedule to assess participant preference for different conditions.

## METHOD

### Participants

Clinicians working at a day and residential program for individuals with IDD were informed about the purpose of the study and invited to nominate individuals with IDD who would potentially benefit from participation. Five individuals who did not have prior experience with SM procedures, who could respond to multiword spoken instructions, and who had increasing independent engagement as an identified goal of instructional programming participated in the study. Prior to participant recruitment, the procedures used were approved by the Western New England University Institutional Review Board. Informed consent for participation was then obtained by a legal guardian for each participant. In addition, prior to all sessions, each participant was invited to participate and independently join the therapist at the table. At any time, if participants indicated that they did not wish to participate or engaged in behavior indicative of not wanting to participate (e.g., crying, moving away from the therapist and table, aggression), the current session was terminated and the next session was postponed.

Chris was a 21‐year‐old young male diagnosed with Fragile X syndrome and intellectual disability. He was nonvocal and communicated using pictures, simple gestures, and manual signs. Caregivers reported that Chris frequently exhibited off‐task behavior (e.g., engagement with nonwork materials) or motor stereotypy when instructed to work or play independently. Chris's individualized education program (IEP) included a specific objective to increase appropriate vocational skills that may facilitate his entry into an employment program.

Bob was a 39‐year‐old male with a diagnosis of autism and intellectual disability. Bob was nonvocal and primarily communicated through the use of a communication book that consisted of written words. Caregivers reported that Bob frequently exhibited off‐task behavior (e.g., engaging with nonwork materials or waiting for assistance from caregivers) when instructed to complete tasks and required additional prompts to complete familiar tasks. Bob's individual support plan included a specific goal to increase independence with exercise and vocational skills.

Scott was a 14‐year‐old male with a diagnosis of autism and intellectual disability. Scott primarily communicated using vocal approximations and gestures. Caregivers reported that he frequently engaged in off‐task behavior or motor stereotypy when instructed to work or play independently. Scott's IEP included objectives for increasing independent leisure item engagement and the completion of prevocational tasks.

Josh was a 20‐year‐old male diagnosed with autism and intellectual disability. He communicated vocally using one‐to‐three‐word sentences. Josh frequently engaged in motor stereotypy (in the form of body rocking and hand flapping) that interfered with completion of educational and vocational tasks. His IEP included goals for increasing independent vocational engagement across vocational tasks.

Blake was a 16‐year‐old male diagnosed with autism and intellectual disability who primarily communicated vocal‐verbally using phrases and full sentences. His caregivers reported that he was frequently distracted and did not consistently remain on task (e.g., he would often engage with other materials in the environment), which limited the independent completion of common daily tasks. Blake's IEP included objectives for completing activities related to developing vocational skills and increasing time spent engaging in vocational tasks.

Chris lived at home and attended a day program for individuals with IDD. Scott, Josh, and Blake lived in a residential facility for individuals with developmental disabilities, and all attended the same day school program as Chris. Bob lived in a residential facility for adults with developmental disabilities. All participants had receptive language skills that allowed them to understand spoken instructions and follow instructions related to task engagement and SM procedures.

### Setting

All sessions occurred in a quiet area of participants' classrooms located within the school (Chris, Scott, Josh, and Blake) or residence (Bob). Each area contained a table, chairs, and relevant task materials. Three to five sessions were conducted once per day, three to five times per week. All sessions were video recorded, and observers subsequently collected data from previously recorded videos.

### Materials

To identify six tasks for inclusion in the task assessment, the experimenter conducted an open‐ended interview with a staff member who served as a clinical supervisor and had worked with the participant for at least 1 year. Tasks were included that (a) the participant was frequently instructed to complete, (b) were available in the participant's school or home, (c) could be placed on a table in front of the participant, and (d) resulted in a series of discrete permanent products. These inclusion criteria were used to ensure that identified tasks could be associated with both engagement and productivity.

For the SM instruction phase, 10‐s video clips of the participant engaging in both on‐task and off‐task behavior were created. Video clips were created using video footage collected during the task assessment for the task used during the treatment component analysis and depicted the participant engaging with the same task materials that were presented in the treatment component analysis. Three to five 10‐s clips of on‐task and off‐task behavior were created for each participant. Video clips were shown to participants on a laptop (Chris, Scott, Josh, and Blake) or desktop computer (Bob).

Supporting Information A includes an example of the custom SM recording sheet created for each participant for use during SM instruction and the treatment component analysis. Each SM recording sheet included two color‐coded columns to facilitate SM. The left column was orange and featured a photograph of the participant engaging in the task, with the word “YES” at the top. Below the photograph, five orange squares provided spaces for the participant to record instances of appropriate task engagement. The right column was blue and displayed a photograph of the participant facing away from the task, with the word “NO” at the top. Below the photograph, five blue squares allowed participants to record instances of off‐task behavior. Each SM recording sheet provided five opportunities per session for participants to self‐monitor their behavior, and multiple copies of the SM recording sheets were provided to the participants each session. During sessions that included SM, the therapist used an app‐based timer on a smartphone to signal to the participant when to self‐monitor.

For all participants, a visual aid was created to support accurate recording. This aid consisted of a cardboard sheet with a cut‐out opening that displayed one row at a time. At the start of each session, the therapist positioned the visual aid over the column of boxes on the SM recording sheet and instructed the participant to move it down one row per trial to ensure alignment with each monitoring opportunity.

Individual reinforcers were selected for each participant. For all participants, a variety of preferred edible items were identified by staff members familiar with the participants' preferences. Additionally, for Chris and Scott, a token board was used as part of their reinforcement system. Both participants had prior experience with a token economy as part of their academic program. They could earn a maximum of 10 tokens per session, which were exchangeable for breaks and access to preferred activities.

### Response measurement and reliability

Data were collected using paper‐and‐pencil recording or the Direct Assessment Tracking Application on a handheld device. Task engagement, measured during the task assessment and treatment component analysis, was measured using 10‐s momentary time‐sampling procedures. Supporting Information B includes a definition of task engagement for each participant–task combination based on observable indicators of active task participation (e.g., sorting materials, placing items into containers, folding laundry). Engagement data were summarized as the percentage of intervals in which the participant was observed to be engaged during each session.

Accurate SM, measured during SM instruction and the treatment component analysis, was defined as the participant placing a mark in the correct column of the SM worksheet at the sound of the tone, based on whether they were engaged in the target behavior (e.g., task engagement) when the tone sounded. Following each SM opportunity, the experimenter compared the participant's response to the experimenter's record. An agreement was scored if both the participant and the experimenter recorded the same response. Accurate SM was summarized as a percentage of opportunities, calculated by dividing the number of agreements by the total number of SM opportunities and multiplying by 100.

Supporting Information B also provides a definition of productivity for each participant and task. Productivity, measured during the task assessment and treatment component analysis, was defined as the number of task‐relevant responses completed per session (e.g., number of items sorted, folded, or matched). Productivity was measured and summarized as responses per minute by dividing the total number of completed responses by the session duration in minutes.

Selection, measured during the treatment preference assessment, was defined as the participant pointing to or vocally stating the color name of one of three colored cards, each associated with a specific treatment condition. Each selection response was recorded as a single frequency count. Selection data were summarized as the cumulative frequency of each condition selected across preference trials.

A second independent observer collected data on all dependent variables for at least 25% of sessions for all phases of the study. Interobserver agreement (IOA) was collected from video‐recorded sessions using paper‐and‐pencil recording or a handheld iTouch device. IOA for engagement was calculated using an interval‐by‐interval agreement method based on 10‐s momentary time sampling. An agreement was scored when both observers recorded the same response (i.e., occurrence or nonoccurrence of engagement) for a given 10‐s interval. IOA was calculated by dividing the total number of intervals with agreement by the total number of scored intervals (agreements plus disagreements) and multiplying by 100 to yield a percentage. IOA for accurate SM was calculated using a per‐opportunity agreement method. Each SM opportunity corresponded to a discrete moment when participants were expected to record their engagement. An agreement was scored if both observers recorded the same SM outcome (YES or NO). IOA was calculated by dividing the number of agreements by the sum of agreements and disagreements and multiplying by 100. IOA for productivity was calculated using a count‐per‐interval agreement method. Observers recorded the number of task‐relevant responses completed in each 1‐min interval. For each interval, agreement was scored by dividing the smaller count by the larger count and multiplying by 100. These percentages were then averaged across all intervals in the session to yield an overall IOA score. IOA for selection during the treatment preference assessment was calculated using a trial‐by‐trial agreement method. An agreement was scored if both observers recorded the same initial‐link selection for a given trial. IOA was calculated by dividing the number of agreements by the total number of trials (agreements plus disagreements) and multiplying by 100. Supporting Information C provides IOA percentages and ranges across participants and phases. Across participants, mean IOA ranged from 89.44% to 99% for engagement during the task assessment, 90% to 99.33% for accurate SM during SM instruction, 94.33% to 96.24% for engagement during the treatment component analysis, 94% to 100% for accurate SM during the treatment component analysis, 90.70% to 99% for productivity during the treatment component analysis, and 100% for initial‐link selection during the treatment preference assessment.

### Procedures

This section provides an overview of the assessment and intervention procedures. Detailed procedural descriptions for each condition are provided in the following section. A task assessment was conducted prior to the treatment component analysis. The purpose of the task assessment was to identify a task that the participant had the skills to complete (Phase 1) but did not consistently do so in the absence of prompting and reinforcement (Phase 2). The task associated with low levels of independent task engagement was selected for each participant and included in the treatment component analysis.

The treatment component analysis was conducted using a reversal design (Riden et al., 2024) to evaluate the independent and combined effects of SM and DR on task engagement and accurate SM. Each session was 5 min. Visual inspection of graphed data was used to evaluate changes in task engagement and accurate SM relative to the preceding phase. Task engagement was considered (a) high if it occurred during 80%–100% of observed intervals, (b) moderate if it occurred during 40%–79% of intervals, and (c) low if it occurred during fewer than 39% of intervals. Similarly, accurate SM was considered (a) high if it occurred in 80%–100% of opportunities, (b) moderate if it occurred in 50%–79% of opportunities, and (c) low if it occurred in fewer than 49% of opportunities. A condition was considered effective when task engagement increased to moderate‐to‐high levels across at least three consecutive sessions. A condition was considered ineffective when task engagement occurred at low levels for at least three consecutive sessions. Conditions that met effectiveness criteria were withdrawn and reintroduced using a reversal design. The control condition used to demonstrate a reversal of behavior change was the previously implemented ineffective condition.

**FIGURE 1:**
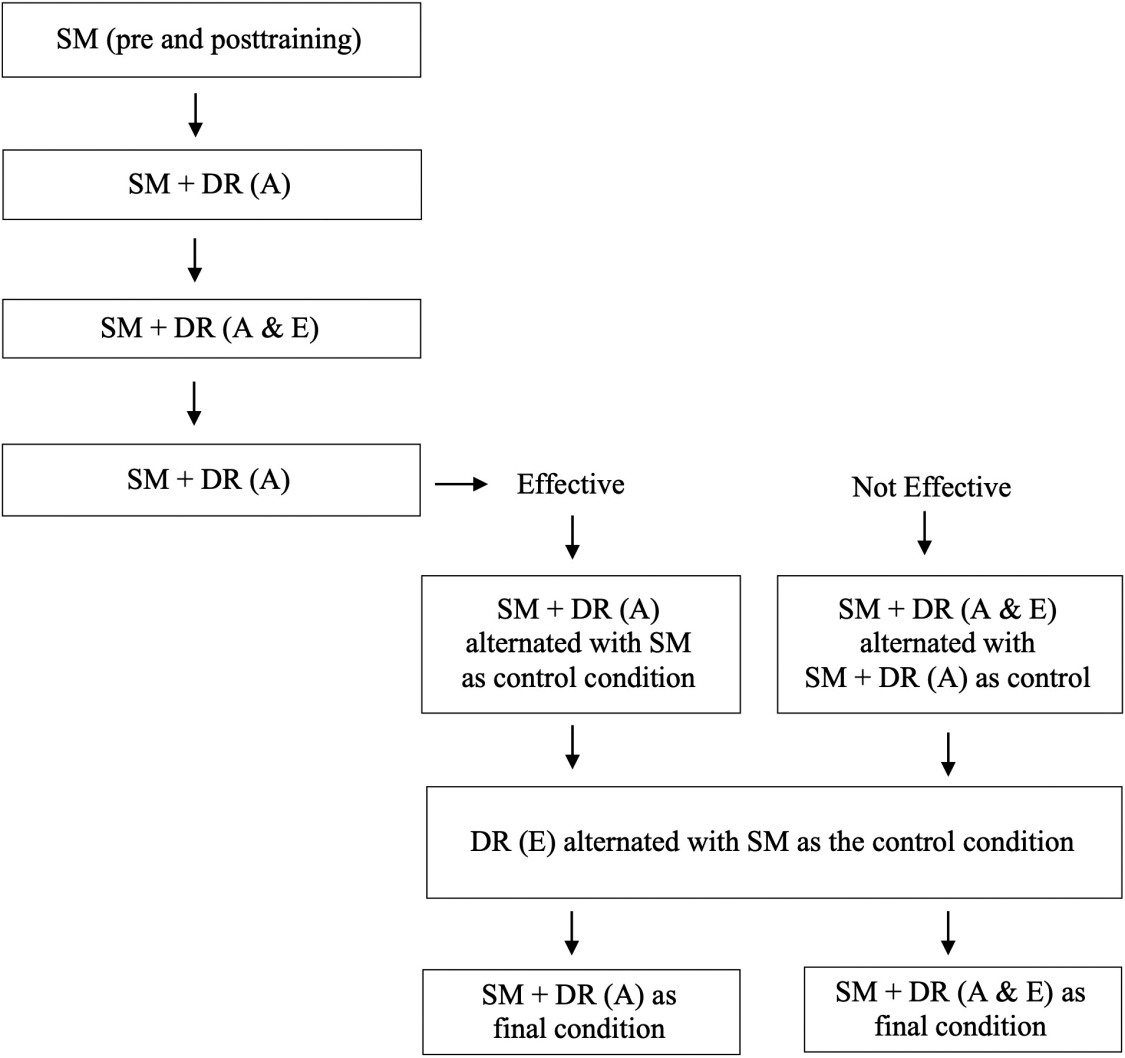
Flowchart depicting the sequences of conditions during the component analysis. SM = self‐monitoring; DR = differential reinforcement of alternative behavior; A = accurate; E = engagement.

Figure 1 illustrates the sequence of conditions conducted during the component analysis. All participants experienced the same initial sequence of conditions: SM (preinstruction), SM instruction, SM (postinstruction), SM + DR (accurate), SM + DR (accurate + engagement), and SM + DR (accurate). At this point, the sequence diverged depending on whether SM + DR (accurate) was effective. For participants for whom SM + DR (accurate) was effective, this condition was alternated with SM as the control condition. For participants for whom SM + DR (accurate) was not effective, SM + DR (accurate) served as the control condition and was alternated with SM + DR (accurate + engagement). For all participants, DR (engagement) was subsequently evaluated and alternated with the SM condition as the control. Finally, the analysis concluded with SM + DR (accurate) if that condition was found to be effective. If not, then the analysis concluded with SM + DR (accurate + engagement). A minor deviation in this sequence occurred for Scott, which is described in the results section.

Finally, a treatment preference assessment was conducted to determine participants' relative preference for the conditions. Due to time constraints, the treatment preference assessment was conducted with only three participants (Bob, Josh, and Blake).

#### Task assessment

Supporting Information D provides a detailed description of the task assessment procedures and results. During Phase 1 of the task assessment, the experimenter presented one of the six tasks identified by each participant's supervisor (see Materials section) for 10 consecutive trials. During each trial, the experimenter presented the task materials and an initial instruction to complete the task (e.g., “Sort the index cards.”). If the participant completed the task independently, the experimenter delivered praise and a small edible chosen by the participant at the start of the session. If the participant did not complete the task or respond within 5 s, the experimenter removed the materials and re‐presented them for the next trial. Tasks that a participant independently completed on 90% or more of trials were included in Phase 2 of the task assessment. During Phase 2 of the task assessment, tasks were alternated in a quasirandom sequence across sessions. Each task was presented individually for three 5‐min sessions. At the start of each session, the experimenter presented one task and corresponding materials and said, “You can do X if you want.” No further prompts or programmed consequences were delivered.

Six tasks associated with at least 90% independent completion were identified for each participant during Phase 1 of the task assessment and were included in Phase 2 (see Supporting Information D). For each participant, one task associated with low levels of task engagement (i.e., <50% engagement, except for Blake, for whom the least engaged task was used) during Phase 2 of the task assessment was selected for inclusion in the treatment component analysis.

#### Self‐monitoring

The SM condition was conducted to establish whether participants would independently engage in tasks and accurately self‐monitor their behavior in the absence of DR. During each session, the participant was seated at a desk with both the task materials and the SM materials. The experimenter provided a neutral instruction at the start of the session: “I have some work for you to do. You can work if you want, but you're not earning anything.” A variable‐momentary (VM) 30‐s timer was used to signal the participant to self‐monitor. No prompts were provided, and there were no programmed consequences for checking boxes or engaging in task‐related behavior.

#### Self‐monitoring instruction

Supporting Information E provides a detailed description of the SM instruction procedures. Following the initial SM phase, the experimenter conducted SM instruction that included two phases, a video phase followed by an in vivo phase. All sessions included 10 trials. During the video phase, the type of performance depicted in the video progressed from occurrence‐only trials to nonoccurrence‐only trials and finally to an interspersal of occurrence and nonoccurrence trials. Once the participant's performance met criteria (i.e., 80% accurate and independent responses across at least two consecutive sessions) with one type of video trial, the experimenter initiated the subsequent video trial type.

During the in vivo phase, sessions consisted of 10 trials that consisted of both occurrence and nonoccurrence trials, similar to interspersed trial video sessions. However, the experimenter sounded a tone 10 times per session to represent each trial. At the sound of each tone, participants were instructed in vivo to self‐monitor whether they did or did not exhibit task engagement. The experimenter contrived naturally occurring learning opportunities by initiating occurrence trials and sounding the tone when the participant displayed task engagement and initiating nonoccurrence trials and sounding the tone when the participant was not displaying engagement.

#### Self‐monitoring + differential reinforcement (accurate)

The purpose of the SM + DR (accurate) condition was to evaluate whether adding reinforcement for accurate SM improved the accuracy of participants' SM and task engagement relative to the SM condition. This condition was similar to the SM condition, except that the DR (accurate) component was introduced. Before the start of each session, participants selected a preferred item that could be earned in exchange for accurate SM. Participants were presented with an array of edibles commonly found in their classrooms or homes (Bob, Josh, and Blake) or pictures of preferred items or activities found in their classroom (e.g., computer games; Chris and Scott). They could indicate their choice either by pointing (Chris, Bob, and Josh) or by vocally stating the item (Scott and Blake). The selected item was then named by the experimenter and used as the reinforcer for that session. At the start of each session, the experimenter said, “I have some work for you to do. When you hear the beep, if you check the correct box, you can have a (name of edible/token).” During each 5‐min session, the timer sounded on a VM 30‐s schedule. The experimenter delivered a preferred edible or token contingent on accurate SM.

For Scott, a procedural variation was introduced during SM + DR (accurate). Scott began refusing the edible when it was delivered contingent on engagement and accurate SM, suggesting that the edible no longer functioned as a reinforcer. We replaced the edible reinforcer with tokens for the remainder of his component analysis on the first session of the second SM + DR (accurate) condition. Given Scott's frequent requests for breaks from various classroom tasks, each token earned a 30‐s break, with a maximum of 10 tokens (equivalent to a 5‐min break) per session.

#### Self‐monitoring + differential reinforcement (accurate + engagement)

The purpose of the SM + DR (accurate + engagement) condition was to evaluate whether adding reinforcement for engagement improved accurate SM and task engagement relative to the SM + DR (accurate) condition. This condition was identical to the SM + DR (accurate) condition, except that both accurate SM and task engagement were required for reinforcer delivery. Additionally, the experimenter modified the instructions to reflect this change in the DR component. At the start of each session, the experimenter said, “I have some work for you to do. When you hear the beep, if you are working and you check the YES box, you can have a (name of preferred edible/token).” During each 5‐min session, the timer sounded on a VM 30‐s schedule. The experimenter delivered praise and a preferred edible or token if the participant exhibited task engagement and accurately recorded the occurrence of their engagement (i.e., correctly recorded the occurrence of their engagement). The participant only received praise and an edible or token if they emitted task engagement and checked the YES box when the timer sounded.

Two procedural variations were introduced for Chris in the first SM + DRA (accurate + engagement) condition. First, a presession exposure prompt was introduced to ensure contact with the reinforcer for task engagement and accurate SM. This exposure included a vocal prompt for Chris to emit task engagement and accurate SM and the delivery of an edible after he emitted both responses. Second, Chris frequently refused the edible when it was delivered contingent on task engagement and accurate SM, suggesting that it no longer functioned as a reinforcer. Because Chris earned tokens as part of his regular reinforcement system, tokens (exchangeable for 30 s of postsession computer time) were subsequently substituted for edibles. He could earn a maximum of 10 tokens for 5 min of computer time.

#### Differential reinforcement (engagement)

The purpose of the DR (engagement) condition was to evaluate the effects of DR for task engagement when the SM component was not in effect. In this condition, the DR contingency was in effect only for task engagement and the SM materials were not presented. However, the timer still sounded on a VM 30‐s schedule. At the start of each session, the experimenter said, “I have some work for you to do. When you hear the beep, if you were working, you can have a (name of preferred edible or token).” The experimenter delivered praise and a preferred edible or token if the participant emitted task engagement when the timer sounded. To compare the effects of access to SM materials versus DR for engagement, this condition was alternated with the SM condition. This allowed for a direct evaluation of whether engagement maintained when SM materials were removed and reinforcement for task engagement remained in place.

#### Treatment preference assessment

The treatment preference assessment included a concurrent‐chain schedule with initial links and terminal links. A paired stimulus preference assessment was first conducted to identify colors that were not highly preferred by the participant. Three different colored cards were affixed to the backs of the chairs (Bob) or placed on a table (Josh and Blake). During the initial link, the participant stood on a piece of masking tape behind the table and chairs. The experimenter prompted the participant to select (point or vocally state the color name) one of the three colored cards. Following the selection, the experimenter instructed the participant to sit at the table. The colored cards were rotated clockwise across trials. The treatment preference assessment continued until differentiated responding was observed (the participant consistently selected one condition) for at least five consecutive trials.

##### No differential consequences

At the beginning of each trial, the experimenter said, “You can choose any of these cards” (initial link). Selection of any card resulted in delivery of an edible. Following the selection, the participant was asked to sit at the corresponding table and the experimenter delivered an additional edible. The participant did not experience any of the treatment conditions, and the trial ended after approximately 30 s.

##### Differential consequences

We randomly assigned one color to each treatment: SM, SM + DR (engagement and accurate), and DR (engagement). At the beginning of each trial, the experimenter said, “You can choose any of these cards” (initial link). The experimenter then explained the contingencies associated with each color. For example, to explain the DR (engagement) contingencies, the experimenter said, “if you choose the red card, you can (do the task and earn X for doing the task), but you don't have to tick any boxes.” Next, the experimenter provided the participant with an opportunity to select one card and sit at the table. After the participant sat down, the experimenter conducted the treatment associated with the selected color for 2 min (terminal link). The task was the same one used in the treatment component analysis.

## RESULTS

Table 1 shows the number of sessions to criterion for accurate self‐monitoring in each phase of self‐monitoring instruction for each participant.

**TABLE 1:** Number of sessions to criterion for accurate self‐monitoring in each phase of self‐monitoring instruction.

Participant	Video instruction			In vivo instruction			Total
	Occurrence	Nonoccurrence	Interspersed	Occurrence	Nonoccurrence	Interspersed	
Chris	4	6	23	6	6	6	51
Bob	4	4	12	NA	NA	11	31
Scott	6	6	24	NA	NA	9	45
Josh	4	4	6	NA	NA	8	22
Blake	6	4	5	NA	NA	4	19

*Note*: NA = not applicable because this phase was not conducted.

**FIGURE 2:**
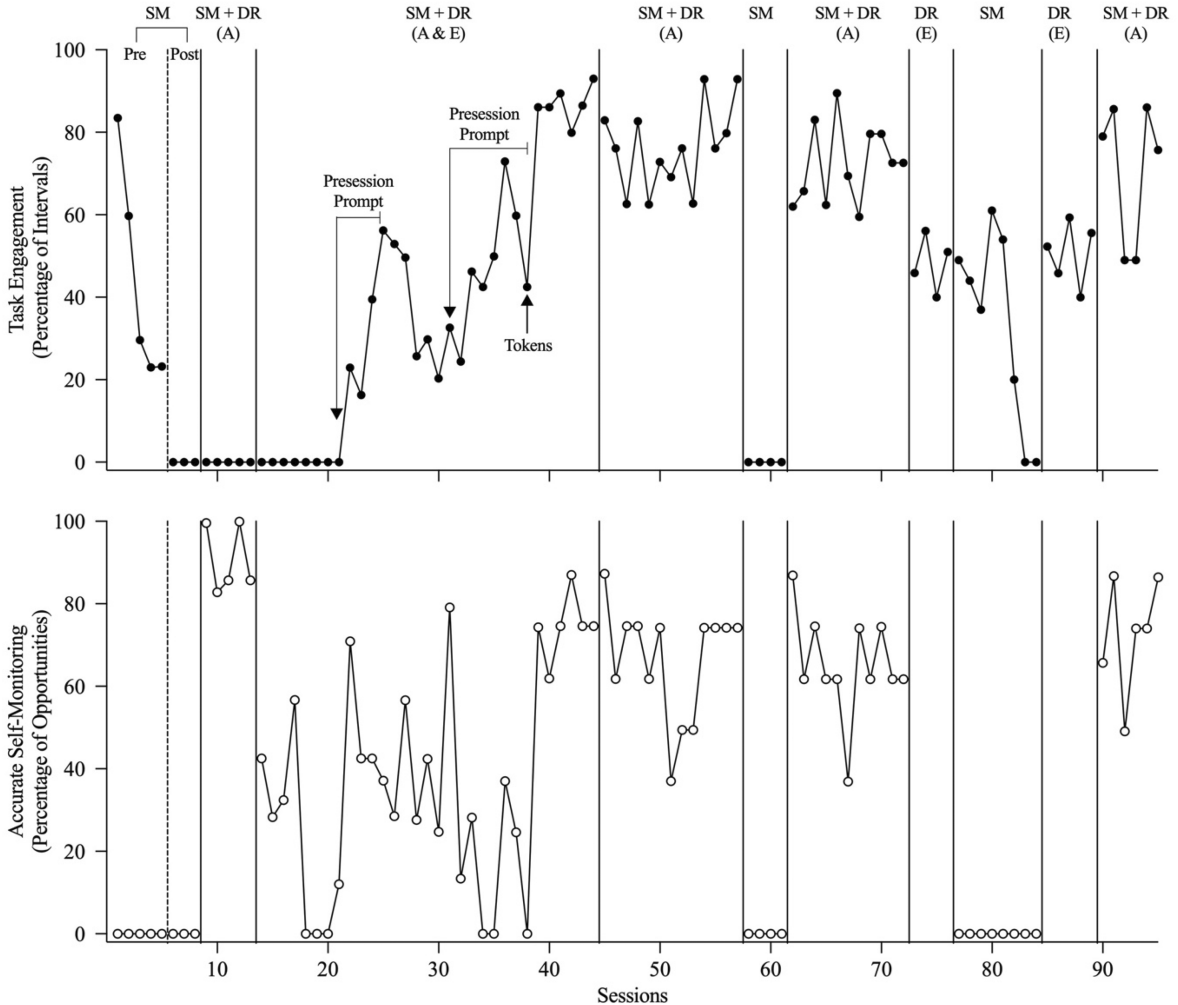
Treatment component analysis for Chris. SM = self‐monitoring; Pre = preinstruction; Post = postinstruction; DR = differential reinforcement of alternative behavior; A = accurate; E = engagement. The dashed line between the preinstruction and postinstruction phases shows when SM instruction was conducted. No data are included in the bottom panel for the DR (E) phase because the participant did not self‐monitor during this condition.

**FIGURE 3:**
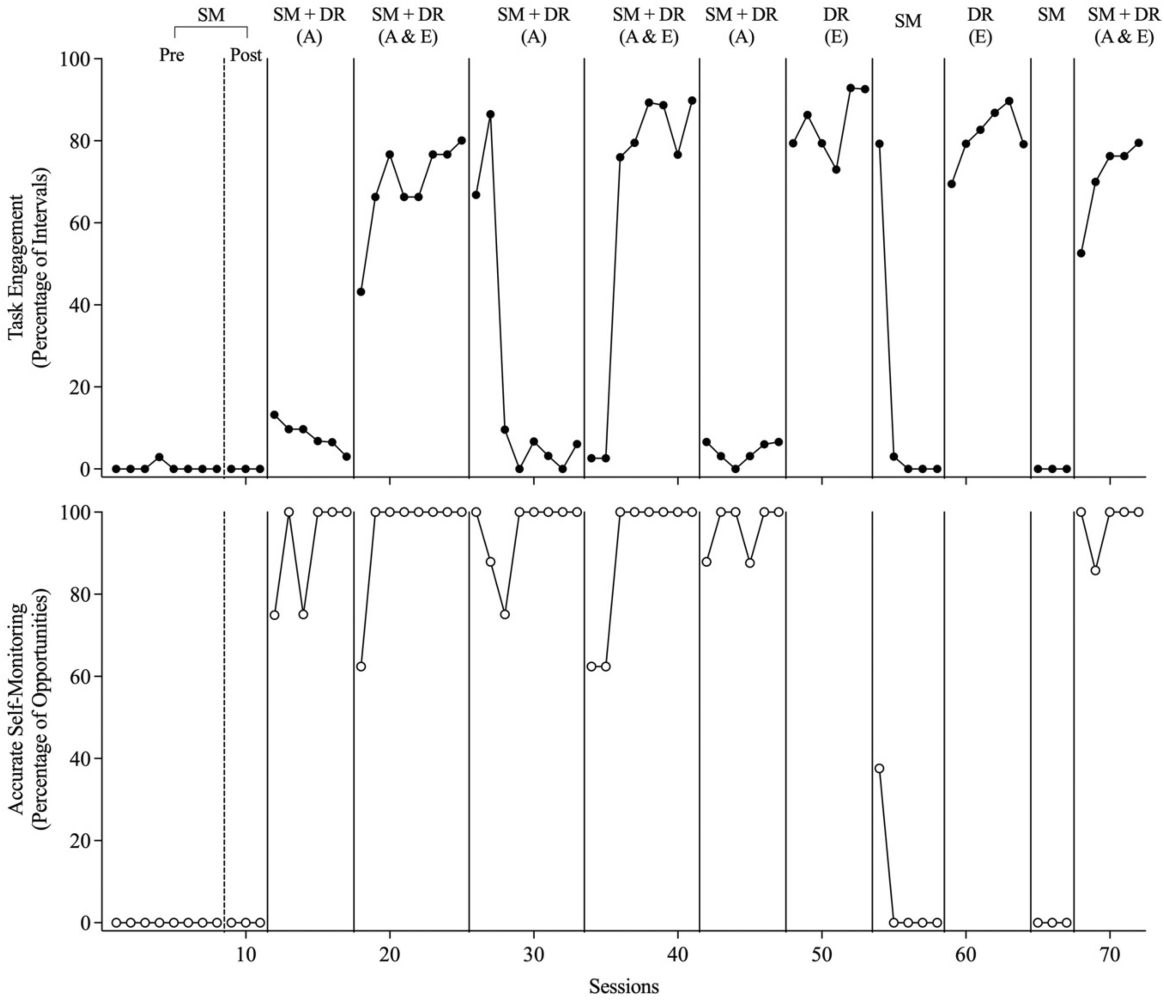
Treatment component analysis for Bob. SM = self‐monitoring; Pre = preinstruction; Post = postinstruction; DR = differential reinforcement of alternative behavior; A = accurate; E = engagement. The dashed line between the preinstruction and postinstruction phases shows when SM instruction was conducted. No data are included in the bottom panel for the DR (E) phase because the participant did not self‐monitor during this condition.

**FIGURE 4:**
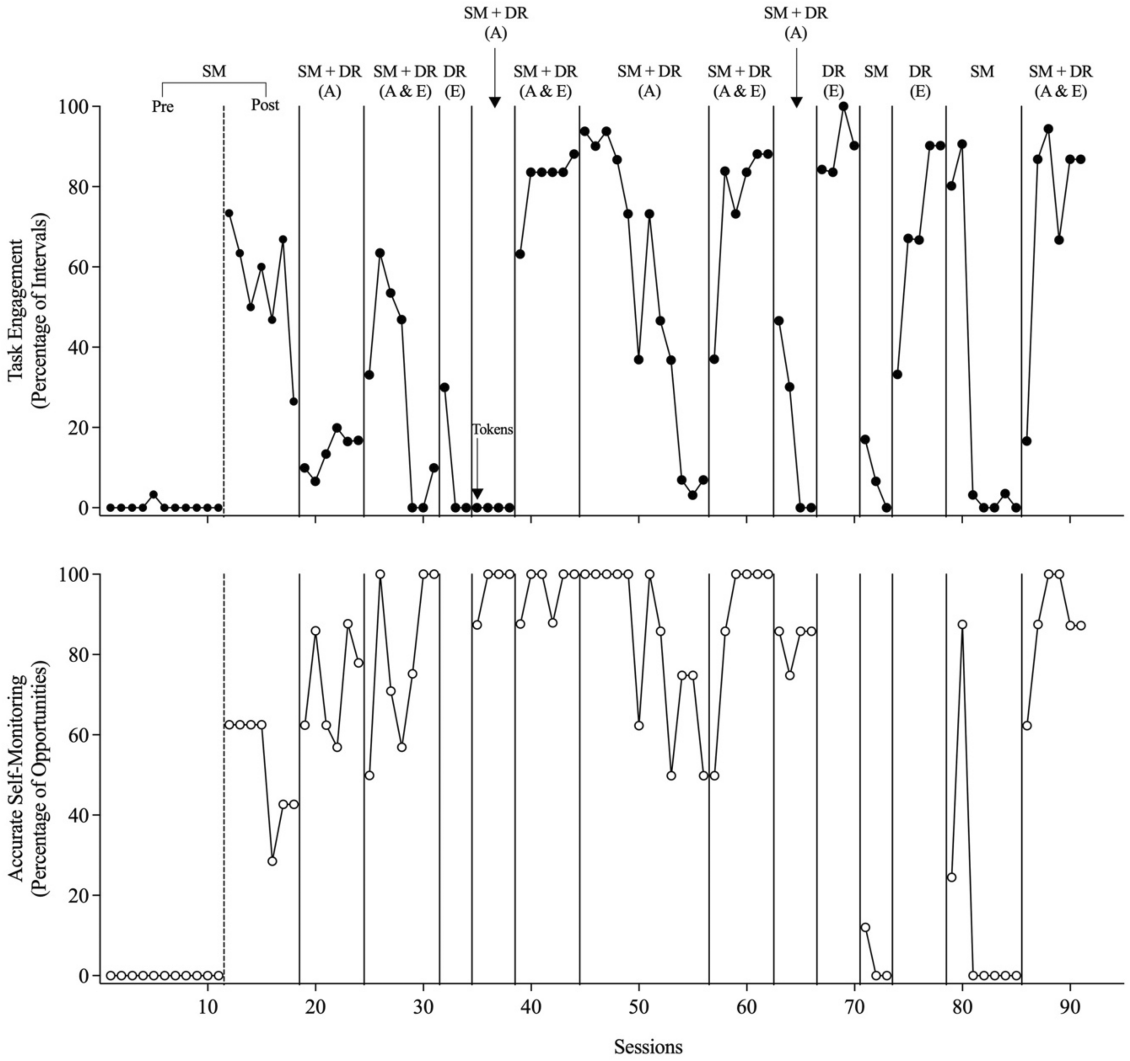
Treatment component analysis for Scott. SM = self‐monitoring; Pre = preinstruction; Post = postinstruction; DR = differential reinforcement of alternative behavior; A = accurate; E = engagement. The dashed line between the preinstruction and postinstruction phases shows when SM instruction was conducted. No data are included in the bottom panel for the DR (E) phase because the participant did not self‐monitor during this condition.

**FIGURE 5:**
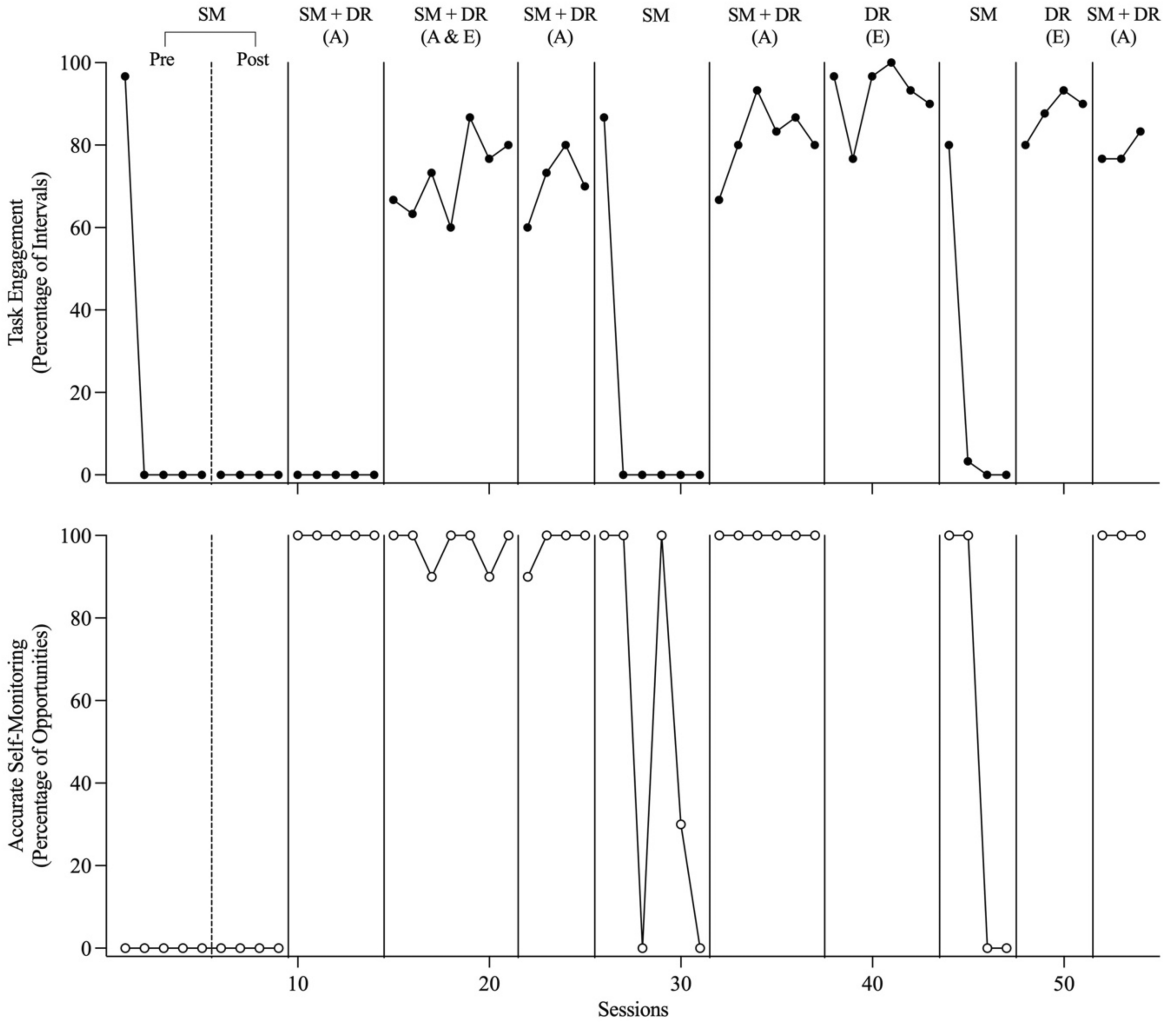
Treatment component analysis for Josh. SM = self‐monitoring; Pre = preinstruction; Post = postinstruction; DR = differential reinforcement of alternative behavior; A = accurate; E = engagement. The dashed line between the preinstruction and postinstruction phases shows when SM instruction was conducted. No data are included in the bottom panel for the DR (E) phase because the participant did not self‐monitor during this condition.

**FIGURE 6:**
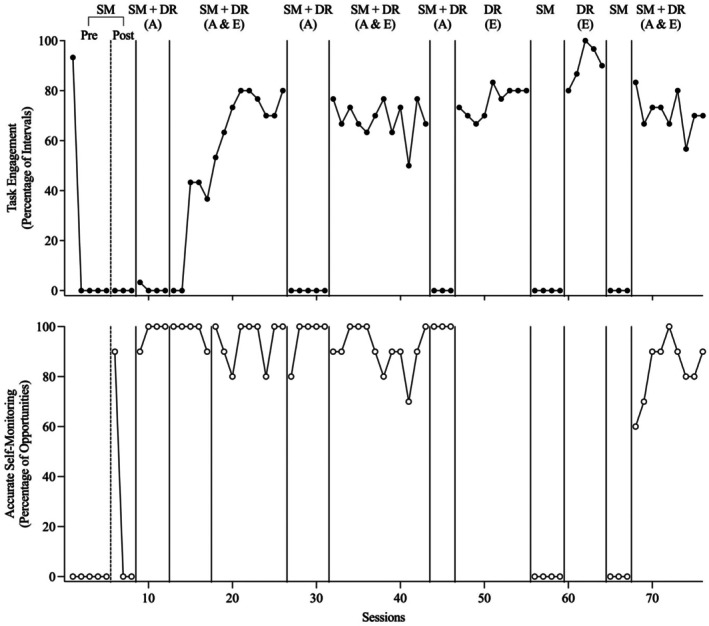
Treatment component analysis for Blake. SM = self‐monitoring; Pre = preinstruction; Post = postinstruction; DR = differential reinforcement of alternative behavior; A = accurate; E = engagement. The dashed line between the preinstruction and postinstruction phases shows when SM instruction was conducted. No data are included in the bottom panel for the DR (E) phase because the participant did not self‐monitor during this condition.

Figures 2, 3, 4, 5, 6 present the results of the treatment component analyses for each of the five participants. Each figure includes two panels. The top panel displays the percentage of intervals with task engagement, and the bottom panel shows the percentage of opportunities with accurate SM.

### Chris

Figure 2 depicts the results of the component analysis for Chris (Figure 2). During the preinstruction SM phase, Chris's task engagement was initially high but rapidly decreased to low levels and accurate SM was not observed. Table 2 and Supporting Information F and G depict the results of SM instruction for each participant. During SM instruction, Chris acquired the SM response in 51 sessions. During the postinstruction SM phase, Chris did not exhibit task engagement or accurate SM, suggesting that SM alone (even after sufficient instruction of accurate SM) was not sufficient to maintain accurate SM or task engagement. During the first SM + DR (accurate) phase, Chris did not exhibit task engagement but accurate SM immediately increased to high stable levels (i.e., Chris accurately self‐monitored his nonengagement).

**TABLE 2:** Responses per minute of productivity (mean) during the last phase for each condition of the treatment component analysis.

Condition	Chris	Bob	Scott	Josh	Blake	Overall mean
SM	2.50	0	1.14	1.82	0	1.09
SM + DR (A)	3.43	0	5.73	10.63	0	3.96
SM + DR (A & E)	3.50	28.96	5.89	7.22	3.33	9.78
DR (E)	3.43	30.63	5.63	14.50	3.90	11.62

*Note*: SM = self‐monitoring; DR = differential reinforcement of alternative behavior; A = accurate; E = engagement.

During the first SM + DR (accurate + engagement) phase, Chris's task engagement initially did not change relative to the preceding phase and accurate SM decreased to variable but lower levels. Following the introduction of the presession exposure prompt, task engagement and accurate SM increased to moderate yet variable levels. However, task engagement did not maintain when the presession prompt was withdrawn (Sessions 26–30) and presession prompting did not sufficiently increase his task engagement when it was reintroduced (Sessions 31–37). When tokens (exchangeable for 30 s of postsession computer time) were subsequently substituted for edibles beginning at Session 38, task engagement and accurate SM rapidly increased. During the second implementation of SM + DR (accurate), task engagement maintained relative to the preceding SM + DR (accurate + engagement) phase and accurate SM occurred at moderate but variable levels suggesting that the SM + DR (accurate) intervention may be effective following an immediate history of SM + DR (accurate + engagement). When the SM condition was reinstated, Chris's task engagement and accurate SM immediately decreased to zero. During the third implementation of SM + DR (accurate), task engagement and accurate SM increased to moderate levels. Levels were similar to those observed in the second SM + DR (accurate) phase, failing to replicate our initial finding that this phase may be ineffective following the SM phase.

Next, we evaluated the effects of DR (engagement) without SM by altering this condition with the SM condition. During DR (engagement), task engagement occurred at moderate levels but levels were not as high as those observed during the previous SM + DR (accurate) phase. The slight decrements in performance may have been due to the withdrawal of the SM materials, which may have functioned as a discriminative stimulus. During the SM phase, in which SM materials were available but there were no programmed consequences for accurate SM or task engagement, accurate SM did not occur and engagement maintained initially and then decreased to zero. When SM + DR (accurate) was implemented a fourth time, at the end of the component analysis, task engagement and accurate SM increased to moderate‐to‐high levels, replicating previously observed levels during this condition.

### Bob

Figure 3 depicts the results of the component analysis for Bob. During the preinstruction SM phase, Bob's task engagement occurred at low stable levels and he did not exhibit accurate SM. During SM instruction, Bob acquired the SM response in 31 sessions. However, in the postinstruction SM phase, Bob did not display task engagement or accurate SM. SM instruction was sufficient to increase accurate SM when reinforcement was provided, but it did not maintain when reinforcement was no longer in effect for accurate SM. During the first SM + DR (accurate) phase, levels of task engagement increased slightly at first but then decreased, although accurate SM immediately increased to high stable levels. Reinforcement of accurate SM increased accurate SM but did not lead to increases in task engagement. During the SM + DR (accurate + engagement) phase, Bob's task engagement immediately increased to moderate‐to‐high levels and accurate SM remained high. When the SM + DR (accurate) phase was implemented a second time, following SM + DR (accurate + engagement), Bob's task engagement maintained at high levels for two sessions but then rapidly decreased to baseline levels. Although Bob's task engagement decreased, accurate SM remained high. When the SM + DR (accurate + engagement) phase was implemented a second time, Bob's task engagement increased and accurate SM remained high. When the SM + DR (accurate) phase was implemented a third time, task engagement immediately decreased to low levels but accurate SM remained high.

Next, the independent effects of DR (engagement) without SM were assessed by alternating this condition with the SM condition. Task engagement immediately increased to high levels during DR (engagement) and rapidly decreased to low levels during the SM condition. During SM, with the exception of one session, Bob did not tick any boxes on the SM data sheet. These findings suggested that, for Bob, DR was the critical component of the intervention. Finally, when SM + DR (accurate + engagement) was implemented again at the end of the component analysis, task engagement immediately increased to moderate levels and accurate SM increased to high stable levels.

### Scott

Figure 4 depicts the results of the component analysis for Scott. During the preinstruction SM phase, Scott's task engagement occurred at near‐zero levels. During SM instruction, Scott acquired the SM response in 45 sessions. During the postinstruction SM phase, Scott's task engagement increased and accurate SM increased to moderate levels. However, there was a decreasing trend in these responses toward the end of the phase. During the first SM + DR (accurate) phase, levels of task engagement were lower than those observed during the second SM phase but accurate SM increased to moderate‐to‐high levels. In the first implementation of SM + DR (accurate + engagement), Scott's task engagement initially increased (for four sessions) but then quickly decreased to low levels, although accurate SM continued to occur at moderate‐to‐high levels. During the second implementation of SM + DR (accurate), Scott displayed zero levels of task engagement but accurate SM increased to high stable levels. Thus, including consequences for accurate SM increased Scott's accurate SM but did not lead to increased task engagement. When edibles were replaced with token reinforcement, Scott's task engagement rapidly increased to moderate‐to‐high levels and accurate SM maintained at high and stable levels. When we returned to SM + DR (accurate), task engagement maintained at high levels for five sessions but then gradually decreased, as did accurate SM. We then reimplemented SM + DR (accurate + engagement) and observed an immediate increase in task engagement and accurate SM, replicating the effects from the previous SM + DR (accurate + engagement) phase. Finally, when SM + DR (accurate) was implemented a fourth time, task engagement rapidly decreased to low levels although accurate SM remained high.

Finally, we assessed the independent effects of DR (engagement) without SM by alternating DR (engagement) and SM. During DR (engagement), task engagement occurred at high levels, and during the SM phase, when reinforcement was withdrawn, task engagement rapidly decreased and accurate SM decreased to low levels. Finally, when SM + DR (accurate + engagement) was implemented again at the end of the component analysis, task engagement and accurate SM increased to high levels.

### Josh

Figure 5 depicts the results of the component analysis for Josh. During the preinstruction SM phase, Josh's task engagement occurred at high levels for one session and then rapidly decreased to zero levels, and he did not exhibit accurate SM. During SM instruction, Josh acquired the SM response in 22 sessions. However, in the postinstruction SM phase, Josh did not display task engagement or accurate SM. During the first SM + DR (accurate) phase, Josh did not display task engagement but accurate SM immediately increased to high levels. During the SM + DR (accurate + engagement) phase, task engagement immediately increased to moderate‐to‐high levels and accurate SM remained high. In the second SM + DR (accurate) phases, Josh's task engagement maintained at moderate‐to‐high levels and accurate SM remained high. When SM was reinstated following the SM + DR (accurate) phase, task engagement occurred at high levels for one session but then decreased to low levels, and accurate SM was variable. When SM + DR (accurate) was implemented a third time, following the SM phase, task engagement immediately increased and accurate SM immediately increased to high levels.

Next, the independent effects of DR (engagement) without SM were assessed by alternating this condition with the SM condition. Task engagement occurred at high, stable levels during DR (engagement) and quickly decreased to low levels during the SM condition. During SM, Josh's accurate SM initially maintained but then rapidly decreased to low levels. Finally, when SM + DR (accurate) was implemented again at the end of the component analysis, task engagement and accurate SM immediately increased to high levels.

### Blake

Figure 6 depicts the results of the component analysis for Blake. During the preinstruction SM phase, Blake's task engagement occurred at high levels for one session and then decreased to zero and he did not exhibit accurate SM. During SM instruction, Blake acquired the SM response in 19 sessions. In the postinstruction SM phase, Blake did not display task engagement and only displayed accurate SM during the first of the three sessions. Similar to the other participants, during the first SM + DR (accurate) phase, Blake did not display task engagement but did display high levels of accurate SM. During the first SM + DR (accurate + engagement) phase, task engagement increased to moderate‐to‐high levels and accurate SM remained high. In the second and third SM + DR (accurate) phases, Blake's task engagement immediately decreased to zero levels, but accurate SM remained high. When SM + DR (accurate + engagement) was implemented a second time following the SM + DR (accurate) phase, task engagement increased to moderate levels and accurate SM maintained at high levels.

When the independent effects of DR (engagement) without SM were assessed, task engagement occurred at moderate‐to‐high levels during both DR (engagement) phases and occurred at zero levels during both SM phases. During SM, Blake's accurate SM also decreased to zero levels. Finally, when SM + DR (accurate + engagement) was implemented again at the end of the component analysis, both task engagement and accurate SM increased to moderate‐to‐high levels.

Table 2 depicts responses per minute of productivity during the last phase for each condition of the component for each participant. Chris and Scott displayed the highest level of productivity in the final SM + DR (accurate + engagement) phase. Bob, Josh, and Blake displayed the highest level of productivity in the final DR (engagement) phase.

### Treatment preference assessment

Supporting Information H depicts the results of the treatment preference assessment of conditions for Bob, Josh, and Blake. During baseline, Bob's initial‐link selections were undifferentiated. Bob selected the middle chair on every trial regardless of the colored card affixed to the back of the chair. During sessions with differential consequences for selection, Bob selected each colored card and experienced each treatment at least one time. However, after trial 15, Bob consistently selected the initial‐link card associated with SM + DR (accurate + engagement), suggesting that he preferred DR combined with SM rather than DR alone.

During the baseline phase, Josh's initial‐link selections were undifferentiated. When there were differential consequences associated with selection of the colored cards, Josh initially selected the initial‐link card associated with DR (engagement). After four sessions, Josh then selected the initial‐link card associated with SM + DR (accurate + engagement) and continued to select this card for the majority of the treatment preference assessment. These data suggested that Josh preferred DR combined with SM rather than DR alone.

Blake's initial‐link selections were undifferentiated during the initial baseline phase. When there were differential consequences associated with selection of the colored cards, Blake consistently selected the initial‐link card associated with DR (engagement). These data suggested that Blake preferred DR without SM.

## DISCUSSION

This study extends previous research on SM by assessing its independent and combined effects for increasing task engagement displayed by individuals with IDD in a component analysis. First, we identified one leisure or vocational task for each participant that they could independently complete but did not complete in the absence of prompting. We then extended the procedures used by Fritz et al. (2012) by teaching participants to accurately self‐monitor their behavior (rather than that of a confederate) during all phases of instruction. In addition, the effects of SM alone (i.e., simply providing SM materials in the absence of reinforcement) was assessed after instruction to determine whether accurate SM was maintained in the absence of reinforcement following instruction. This differed from Fritz et al., who did not conduct SM alone; SM was always conducted with DR for accurate SM. Although all five participants learned to independently and accurately self‐monitor their task engagement during video and in vivo instruction sessions when reinforcement was delivered for accurate SM, none of the participants exhibited task engagement or accurate SM during the postinstruction SM alone condition. For all participants, when SM was combined with DR for accurate SM and task engagement, both accurate SM and task engagement increased. DR (engagement) without SM resulted in similarly high levels of task engagement for all participants.

### Does task engagement and accurate SM maintain following SM instruction?

The first goal of this study was to identify whether the presence of SM materials alone would increase or maintain task engagement or accurate SM following SM instruction. The findings suggest that the presence of SM materials, even when used with an auditory prompt (sound of a timer), was not sufficient to evoke independent engagement or accurate SM. This contrasts with some prior literature suggesting SM may function as a discriminative stimulus (Looney et al., 2018) or response prompt (Kolbenschlag & Wunderlich, 2021) and indicates that additional components (e.g., reinforcement) may be needed.

An extension of previous research was that we conducted a comprehensive SM instruction procedure with multiple phases (with both video and in vivo trials) prior to the component analysis. By ensuring that participants could accurately record their own behavior from video and in vivo before the treatment analysis, we minimized the likelihood that decreases in accurate SM were due to a skill deficit. In the current study, all participants reached criterion levels of accurate SM during instruction, confirming that they acquired the necessary discrimination skills. However, when the SM condition was repeated postinstruction, again without reinforcement, participants did not engage in tasks or accurately self‐monitor their behavior. These results indicate that although SM instruction produced accurate SM, the effects did not maintain postinstruction. The failure of SM effects to maintain following instruction may be due to the absence of reinforcement during the SM alone condition rather than a skill deficit. Although participants acquired the discrimination skills necessary for accurate SM during instruction (including both video and in vivo trials), the lack of contingent consequences in the postinstruction context may have reduced the likelihood that participants would engage in tasks or continue accurate SM. This suggests that acquisition of SM skills alone may be insufficient for maintenance and that ongoing reinforcement may be necessary to sustain the effects.

### Does SM + DR (accurate) or SM + DR (accurate + engagement) increase task engagement and accurate SM?

The second goal of this study was to evaluate whether the addition of differential reinforcement for accurate SM or task engagement and accurate SM alone would increase task engagement and accurate SM. For all participants, the initial implementation of SM + DR (accurate) increased accurate SM but not task engagement (i.e., participants accurately self‐monitored their nonengagement). For Chris and Josh, both accurate SM and task engagement occurred at high levels during SM + DR (accurate) when this condition was preceded by SM + DR (accurate + engagement). These results suggest that SM + DR (accurate) may be sufficient to maintain high levels of task engagement following a previous history of DR for engagement. Another plausible explanation is that SM + DR (accurate) was only effective for Chris once an appropriate reinforcer was identified and delivered through the token economy. In contrast, for the remaining three participants, task engagement did not maintain when SM + DR (accurate) was reintroduced following the SM + DR (accurate + engagement) phase. For these participants, the DR contingency for task engagement was necessary for sustaining high levels of task engagement.

### Does SM enhance the effects of DR (alone) for task engagement?

The third goal of this study was to evaluate whether SM enhanced the effects of DR for task engagement. To this end, we alternated the DR (engagement) and SM conditions to evaluate the independent effects of DR and SM. Notably, some participants demonstrated sustained engagement when reinforcement was delivered contingent on task engagement alone, whereas others required the additional presence of SM materials to maintain higher levels of engagement. For example, Chris' engagement decreased when the SM component was removed during the DR (engagement) condition, whereas Bob, Scott, Josh, and Blake showed comparable or even increased engagement levels under DR (engagement) alone. These findings suggest that the inclusion of SM may enhance the effectiveness of reinforcement‐based interventions for some individuals but may be unnecessary for others. In the current study, SM + DR (accurate) was only effective for two of the five participants after they had been exposed to SM + DR (engagement + accurate), suggesting that prior experience with reinforcement for engagement may support the later effectiveness of more targeted SM procedures.

These findings raise an important conceptual consideration. For SM to be effective, individuals may need to not only accurately observe and record their own behavior but also contact meaningful reinforcement for engaging in SM. In conditions where reinforcement was delivered solely for accurate SM, some participants may have learned that task engagement itself was not associated with programmed consequences, potentially resulting in lower levels of task engagement. In this context, the availability of SM materials may have served primarily as discriminative stimuli signaling when SM should occur rather than SM coming under the control of reinforcement contingencies that would strengthen task engagement.

However, some evidence suggests that for certain participants, SM may have become part of a response chain that was strengthened by the reinforcement produced by high levels of task engagement. For example, both Chris and Josh demonstrated high levels of task engagement in the SM + DR (accurate) condition despite reinforcement being delivered only for accurate SM. For Chris, task engagement was higher in the SM + DR (accurate) condition than in the DR (engagement) condition, suggesting that the SM response (e.g., checking the “yes” box) or materials may have functioned as conditioned reinforcers that helped maintain engagement in the absence of direct reinforcement for that behavior. This interpretation aligns with prior research (e.g., Ardoin & Martens, 2004), which showed that participants may engage in consistent behavioral patterns—either high or low—to maximize accurate SM, even in the absence of naturally occurring reinforcement. These findings highlight the potential for SM to function not only as a monitoring tool but also as part of a response chain in which observing and recording one's own behavior may serve as discriminative stimuli that occasion subsequent task engagement, depending on the individual and their reinforcement history.

### Do participants show a preference for intervention conditions that include SM?

The fourth goal of this study was to assess participant preference for SM. Because more than one effective condition was identified for each participant, we conducted a treatment preference assessment for three participants (Bob, Josh, and Blake) to assess which condition they preferred. Bob and Josh consistently chose the card associated with SM + DR (accurate + engagement), indicating a preference for a condition that included SM. In contrast, Blake consistently chose the card associated with DR (engagement), suggesting that he preferred reinforcement for engagement without the SM component. These findings illustrate that preference assessments can be used to identify participants' preferred treatment option when more than one potentially effective treatment is identified (e.g., SM + DR or DR alone).

### Limitations and future research

This study demonstrates the independent and combined effects of SM and DR on independent task engagement and accurate SM for individuals with IDD. However, several limitations should be considered when interpreting the findings. First, in some cases, adjustments to reinforcers were made during the treatment component analysis. Although these decisions were necessary to identify effective treatments for Bob and Scott, they were not planned a priori and were not implemented consistently across all participants. This highlights the importance of conducting thorough individualized preference or reinforcer assessments prior to intervention to ensure that reinforcement contingencies are sufficiently motivating from the outset. In addition, future research should consider the use of more naturally occurring or delayed reinforcers that may be less likely to interfere with task engagement and that address potential concerns associated with the use of edible reinforcement.

Second, contingency‐specifying instructions were provided to participants at the start of each condition. This instruction may have inadvertently signaled to participants that engagement was or was not expected or required in the SM + DR (accurate) condition. As a result, lower levels of engagement may have been influenced by the instruction. Future research could address this limitation by systematically evaluating the effects of different types of task instructions during conditions with and without SM or DR for engagement or accurate SM.

Third, procedural fidelity was not systematically measured, limiting certainty that procedures were implemented consistently across sessions and participants. Future research should include formal procedural fidelity measures to ensure reliable implementation and strengthen the internal validity of findings. Fifth, although SM + DR (accurate) was effective for two participants and SM + DR (accurate + engagement) was effective for three participants, we did not attempt to fade reinforcement schedules. The study concluded with relatively dense reinforcement schedules. Future research could examine whether the SM component functions as a conditioned reinforcer and facilitates DR schedule thinning, comparing DR alone with SM + DR for maintaining task engagement under thinner schedules.

Future research should evaluate transfer of skills across tasks, settings, or materials; investigate whether generalization occurs spontaneously or requires additional programming (e.g., multiple‐exemplar instruction, varied task practice); and include follow‐up assessments at extended intervals to examine the durability of treatment effects.

## AUTHOR CONTRIBUTIONS

The first author conceptualized the study, conducted the experiment, analyzed the data, drafted the initial manuscript, and contributed to revisions of the manuscript. The second author contributed to conceptualization, methodology, supervision of the project, and completed revisions of the manuscript. The third and fourth authors assisted with the experiment and revisions of the manuscript.

## CONFLICT OF INTEREST STATEMENT

There is no conflict of interest to declare.

## ETHICS APPROVAL

This study received Institutional Review Board approval and was conducted in accordance with established ethical guidelines for the treatment of human participants.

## Supporting information


**Data S1.** Supporting Information

## Data Availability

The data that support the findings of this study are available from the corresponding author upon reasonable request. Supporting Information A includes an example of the self‐monitoring data sheet used by participants. Supporting Information B includes operational definitions for appropriate task engagement and productivity. Supporting Information C includes the interobserver agreement data. Supporting Information D includes the task assessment procedures and data. Supporting Information E includes a description of the SM instruction procedures and Supporting Information F includes a narrative description of the SM instruction results. Supporting Information G includes the self‐monitoring instruction data. Supporting Information H includes the treatment preference assessment data.
