# Supplementary material for: Component analysis of a self‐monitoring intervention for increasing task engagement for individuals with developmental disabilities
Source: J Appl Behav Anal. 2026 Feb 6;59(2):e70053. doi: 10.1002/jaba.70053 (PMC12879527; doi:10.1002/jaba.70053)
Supplement: Supplementary file 1 — Data S1. Supporting Information [file JABA-59-0-s001.docx]

**Supporting Information**

**Supporting Information A**

*Copy of the Self-Monitoring Recording Sheet for Chris*


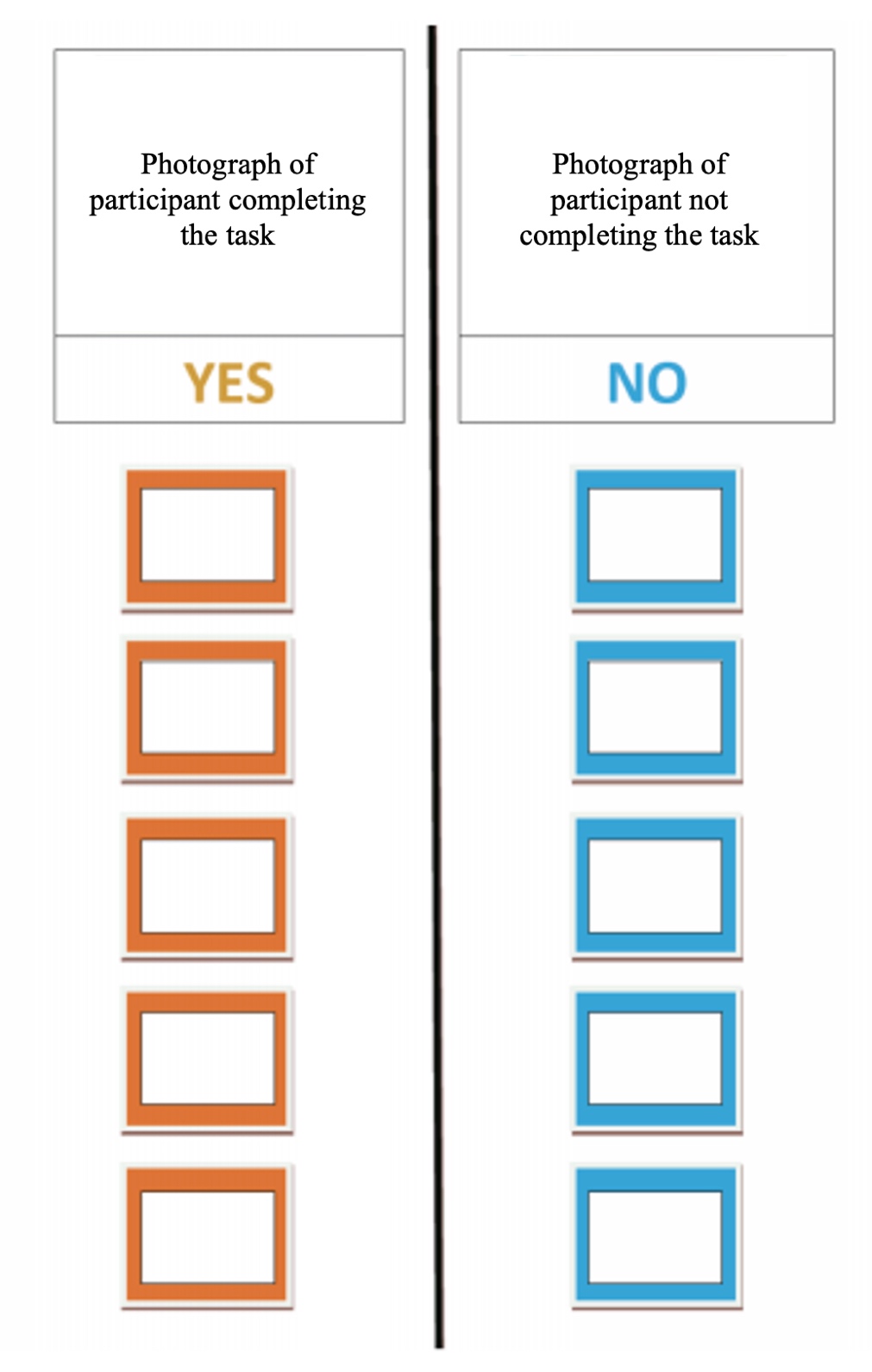


**Supporting Information B**

*Operational Definitions of Appropriate Task Engagement and Productivity*

| Task | Appropriate Task Engagement | Productivity |
| --- | --- | --- |
| Beading | Pick up bead from the pile, pick up string, thread string through bead opening, and push bead to the end of the string. | Total number of beads successfully strung onto the string. |
| Filing Cards | Pick up index card from the pile, flip through the filing box, and file index card behind the correct color tab. | Total number of index cards correctly filed into their respective color tab. |
| Building Legos | Pick up a Lego piece from the bin or pile, stack it onto the baseboard or on top of another Lego piece. | Total number of Legos successfully stacked or assembled. |
| Completing Math Facts | Orient toward the computer screen, scan the math fact, and select the correct answer by clicking. | Total number of math facts completed. |
| Assembling Puzzle | Pick up a puzzle piece from the bin or pile, scan or arrange pieces on the table, attempt to fit or fit the pieces together. | Total number of puzzle pieces successfully fitted together. |
| Assembling Nuts and Bolts | Pick up nut and bolt from separate piles, align bolt with corresponding nut, twist nut onto bolt. | Number of nuts and bolts assembled. |
| Sorting Money | Pick up a bill or coin from the pile, match the bill or coin to the correct envelope, and place the bill or coin inside the correct envelope. | Total number of bills or coins correctly sorted into the appropriate envelopes. |
| Sorting Silverware | Pick up a utensil, match it to the correct row, and place the utensil in the tray. | Total number of utensils successfully sorted into the correct row in the tray. |
| Stamping | Pick up a stamp, press it onto the ink pad, and then press the stamp onto paper to create a design. | Total number of designs successfully stamped onto the paper. |
| Stenciling | Pick up the stencil, position it on the paper, pick up a pencil, and stencil the letter on the paper. | Total number of letters or shapes successfully stenciled. |
| Stuffing Envelopes | Pick up a letter, fold the letter, pick up an empty envelope, place the folded letter inside the envelope, and seal the envelope. | Total number of envelopes successfully stuffed with letters. |
| Weight Lifting | Pick up one weight in each hand, perform various movements (e.g., bicep curls, shoulder presses, lateral raises, or overhead presses), and lower weights to starting position after each repetition. | Total number of repetitions of the lifting movement completed. |
| Collating | Pick up set of papers, arrange them in order, staple or bind them together. | Number of sets of papers collated. |
| Silverware Wrapping | Pick up utensil(s), place it in a napkin, fold napkin around utensil(s), place wrapped utensil(s) in storage container. | Number of pieces of silverware wrapped. |
| Clothes Folding | Pick up piece of clothing, fold clothing along designated creases, place folded item in storage area. | Number of clothing items folded. |
| Coffee Stocking | Pick up coffee bags, place them on shelves, arrange them neatly by type or size. | Number of coffee bags stocked. |
| Table Cleaning | Pick up dirty dishes and items from table, wipe down surface, and place cleaned items in designated storage or disposal area. | Number of tables cleaned. |
| Shelf Stocking | Pick up items from storage area, arrange items on shelves, ensure they are organized by type or size. | Number of items stocked on shelves. |

**Supporting Information C**

*Interobserver Agreement Percentages and Ranges Across Participants and Phases*

| **Participant** | **Phase** | **Behavior** | | **Mean (Range)** |
| --- | --- | --- | --- | --- |
| Chris | Task Assessment | Engagement | 97% (90%–100%) | |
|  | SM Instruction | Accurate SM | 99% (75%–100%) | |
|  | Treatment Component Analysis | Engagement | 94.62% (90%–100%) | |
|  |  | Accurate SM | 94.0% (75%–100%) | |
|  |  | Productivity | 98.62% (96.72%–100%) | |
| Bob | Task Assessment | Engagement | 99% (96.72%–100%) | |
|  | SM Instruction | Accurate SM | 98% (93.33%–100%) | |
|  | Treatment Component Analysis | Engagement | 96.24% (86.72%–100%) | |
|  |  | Accurate SM | 97.84% (87.50%–100%) | |
|  |  | Productivity | 92% (80.90%–96.72%) | |
|  | Treatment Preference Assessment | Initial Link Selection | 100% | |
| Scott | Task Assessment | Engagement | 91% (80%–96.72%) | |
|  | SM Instruction | Accurate SM | 90% (62.50%–100%) | |
|  | Treatment Component Analysis | Engagement | 95.72% (80%–100%) | |
|  |  | Accurate SM | 98.20% (87.50%–100%) | |
|  |  | Productivity | 99% (96%–100%) | |
| Josh | Task Assessment | Engagement | 89.44% (72.60%–100%) | |
|  | SM Instruction | Accurate SM | 95.62% (80%–100%) | |
|  | Treatment Component Analysis | Engagement | 94.33% (86.90%–100%) | |
|  |  | Accurate SM | 100% | |
|  |  | Productivity | 90.70% (72%–100%) | |
|  | Treatment Preference Assessment | Initial Link Selection | 100% | |
| Blake | Task Assessment | Engagement | 90.42% (78.74%–100%) | |
|  | SM Instruction | Accurate SM | 99.33% (90%–100%) | |
|  | Treatment Component Analysis | Engagement | 95.20% (83.70%–100%) | |
|  |  | Accurate SM | 100% | |
|  |  | Productivity | 92.42% (76.20%–100%) | |
|  | Treatment Preference Assessment | Initial Link Selection | 100% | |

*Note.* SM = self-monitoring.

**Supporting Information D**

*Description of the Task Assessment Procedures*

The purpose of the task assessment was to identify a task that each participant had the skills to complete independently (Phase 1) but did not consistently do so in the absence of prompting or reinforcement (Phase 2). Tasks were identified through an indirect assessment, which included caregiver interviews and a review of educational or behavioral programming materials. Based on this information, six leisure or vocational tasks were selected for each participant. These tasks were familiar to the participant, required materials that were readily available in the participant’s classroom or home, and could be completed independently within a short time frame (e.g., sorting index cards, wiping down a table, placing items in bins). The task assessment was conducted across two phases. Only one task was assessed at a time in both phases, with all other materials removed or out of reach to minimize distraction.

**Phase 1: Skill Verification**

During this phase, each of the six tasks was assessed across 10 consecutive trials, with one task assessed per session. At the beginning of each trial, the experimenter presented the relevant materials and delivered a clear instruction to begin the task (e.g., “Sort the index cards.”). The participant had up to 5 s to initiate the task independently. If the participant completed the task without assistance, the experimenter immediately delivered verbal praise (e.g., “Nice job sorting!”) and a small edible item chosen by the participant at the beginning of the session. If the participant did not engage in the task or did not complete it within 5 s, the experimenter removed the materials without comment and initiated the next trial. This process continued until all 10 trials for a given task were completed. The same procedure was repeated for each of the six tasks, with the order of task presentation counterbalanced across participants. Tasks that were completed independently in at least 90% of trials were identified as mastered and selected for inclusion in Phase 2.

**Phase 2: Assessment of Task Engagement in the Absence of Prompts and Reinforcement**

In Phase 2, the tasks that met the 90% accuracy criteria in Phase 1 were assessed under baseline conditions. Each task was presented one at a time in a quasi-random sequence across sessions, such that no task was repeated in back-to-back sessions and each was presented for a total of three 5-min sessions. At the start of each session, the experimenter placed the materials associated with a single task on the table and provided a neutral statement (e.g., “You can do [task] if you want to.”). No further prompts, feedback, or reinforcement were delivered. Task engagement and productivity were observed and recorded, with particular attention to whether the participant engaged in the task independently and for how long.

Across participants, all six tasks met the mastery criterion in Phase 1. However, engagement during Phase 2 varied by task. For each participant, one task associated with low or inconsistent levels of task engagement and productivity in Phase 2 was selected for inclusion in the treatment component analysis, as it met the criteria of being a known task that the participant could complete independently but did not reliably do so without additional prompts.

*Percent Accurate Task Completion and Task Engagement and Responses Per Minute of Productivity for Each Task Presented During Phase 1 and Phase 2 of the Task Assessment*

| Participant | Task | % Accurate  (Phase 1) | % Engagement  (Phase 2) | RPM Productivity  (Phase 2) |
| --- | --- | --- | --- | --- |
| Chris | Assembling nuts & bolts | 100 | 62 | 0 |
|  | Silverware sorting | 100 | 39 | 6.70 |
|  | Envelope stuffing | 100 | 39 | 0 |
|  | Stringing beads* | 100 | 27 | 2.78 |
|  | Index card sorting | 100 | 84 | 14 |
|  | Sorting coins | 92 | 22 | 3.10 |
| Bob | Stencilling | 100 | 52 | 6.30 |
|  | Assembling nuts & bolts | 100 | 20 | 4.22 |
|  | Beading | 100 | 63 | 10.22 |
|  | Weightlifting* | 100 | 11 | 1.80 |
|  | Stamping | 100 | 12 | 4.18 |
|  | Filing Cards | 90 | 58 | 9.60 |
| Scott | Assembling nuts & bolts | 100 | 62.42 | 0 |
|  | Assembling puzzles | 100 | 58.30 | 2 |
|  | Building Legos | 100 | 37.79 | 4.90 |
|  | Completing math facts | 100 | 30 | 6.74 |
|  | Index card sorting | 100 | 47.30 | 12.8 |
|  | Stringing beads* | 93 | 37.56 | 1.56 |
| Josh | Collating | 100 | 28 | 4.40 |
|  | Silverware sorting | 100 | 33 | 7.56 |
|  | Envelope stuffing* | 100 | 33 | 2.22 |
|  | Silverware wrapping | 100 | 0 | 0 |
|  | Index card sorting | 100 | 0 | 0 |
|  | Shelf stocking | 100 | 0 | 0 |
| Blake | Clothes folding* | 100 | 51 | 1.80 |
|  | Coffee stocking | 100 | 74 | 1.84 |
|  | Envelope stuffing | 100 | 80 | 1.64 |
|  | Silverware wrapping | 100 | 78 | 1.08 |
|  | Table cleaning | 100 | 89 | 0.82 |
|  | Shelf stocking | 100 | 92 | 4.60 |

*Note.* RPM = responses per minute

* Indicates task selected for inclusion in the treatment component analysis

**Supporting Information E**

*Description of the Self-Monitoring Instruction Procedures*

The purpose of this condition was to teach the participant to accurately and independently self-monitor their behavior. Following the preinstruction self-monitoring (SM) baseline condition, the experimenter conducted a systematic instruction of SM that included two phases, a video phase followed by an in-vivo phase. All sessions included 10 trials.

**Video Instruction**

During the video phase, the type of performance depicted in the video progressed from occurrence-only trials to nonoccurrence-only trials and finally to an interspersal of occurrence and nonoccurrence trials. Once the participant’s performance met criteria (i.e., 80% accurate and independent responses across at least two consecutive sessions) with one type of video trial, the experimenter initiated the subsequent video trial type.

Prior to the start of each video instruction session, the experimenter provided the instruction “*I have a video for us to watch. At the end of the video, you will need to check a box. If you saw yourself working in the video, you will need to check the “YES” box (the experimenter pointed to the picture of the participant working appropriately on the SM sheet). And if you saw yourself not working in the video, you will need to check the “NO” box (the experimenter pointed to the picture of the participant not working on the SM sheet). If you check the correct box, you will get a (name of preferred edible).*

During occurrence only trials, the experimenter played a 10-s video of the participant engaging with the task appropriately (occurrence) during each trial. Three to five video clips were randomly interspersed during video instruction sessions. For example, the experimenter showed the participant a video of himself sorting index cards correctly for 10 s. At the end of each 10-s video clip, a tone sounded, and the experimenter provided a vocal prompt (“check the YES box”) and simultaneously pointed to the correct box to be checked on the SM data sheet. The experimenter provided praise and a preferred edible for independent and prompted correct responses. Vocal and point prompts were systematically faded in 2-s increments after two consecutive sessions with 80% accuracy. If the participant responded incorrectly, the experimenter erased the incorrect check and then prompted the correct response using a vocal prompt (“you were working, so you can check the YES box”) and point prompt. Praise and a preferred edible were not provided after the prompted response during the error correction. During nonoccurrence trials, the procedures were identical to those used during the occurrence trials, except that the experimenter played a 10-s video clip depicting the participant not engaging in the task (nonoccurrence) during each trial. During interspersed trials, the procedures were identical to those used during the occurrence trials, except that the experimenter varied the type of 10-s video clip (occurrence or nonoccurrence) presented across trials such that each trial type was presented five times in a random order.

**In Vivo Instruction**

During the in vivo phase, sessions consisted of 10 trials that consisted of both occurrence and nonoccurrence trials, similar to interspersed trial video sessions. However, the experimenter sounded a tone 10 times per session to represent each trial. At the sound of each tone, participants were instructed in vivo to self-monitor whether they did or did not exhibit engagement. The experimenter contrived naturally occurring learning opportunities by initiating occurrence trials and sounding the tone when the participant was displaying engagement and initiating nonoccurrence trials and sounding the tone when the participant was not displaying engagement. In each session, the therapist randomly alternated between trial types such that a total of five each of nonoccurrence and occurrence trials were conducted. The experimenter determined the randomization prior to each session by using a random number generator. The random number generator calculated a series of 10 numbers ranging from 1 to 2 and no more than two of the same number occurred in a row. A “1” represented an occurrence trial and a “2” represented a nonoccurrence trial. The experimenter then waited for the participant to display an occurrence or nonoccurrence of engagement depending on the pre-determined trial type.

Before each in vivo instruction session, the therapist stated, “*If you are working when you hear the beep, you will need to check the “YES” box (the experimenter pointed to the picture of the participant working appropriately on the SM sheet). And if you are not working when you hear the beep, you will need to check the “NO” box (the experimenter pointed to the picture of the participant not working on the SM sheet). If you check the correct box, you will get a (name of preferred edible).”* At the sound of the tone, the experimenter presented a vocal and point prompt to the correct box to be checked on the SM data sheet. The experimenter provided praise and a preferred edible for independent and prompted correct responses. Vocal and point prompts were systematically faded in 2-s increments after two consecutive sessions with 80% accuracy. If the participant responded incorrectly, the experimenter erased the incorrect check and then prompted the correct response (praise and edible reinforcer were withheld). Instruction was complete when the participant demonstrated 80% accuracy and independence across at least two consecutive sessions during the in vivo phase. Once the participant met the mastery criterion during in-vivo instruction, the reinforcement component was removed to assess the effects of presenting the SM materials alone (i.e., the first SM baseline phase was conducted).

**Supporting Information F**

*Results of Self-Monitoring Instruction for Chris, Bob, Scott, Josh, and Blake*

Supporting Information G depicts the results of SM instruction for Chris. Chris quickly acquired the SM response during occurrence only and nonoccurrence only trials. When occurrence and nonoccurrence trials were interspersed, Chris’s performance remained variable without improvement, and he was observed checking the boxes on the SM datasheet out of sequence. Therefore, the experimenter placed the visual aid over the column of boxes on the SM datasheet and instructed Chris to move down the opening of the visual aid by one row so that it corresponded with each trial. Following this modification, Chris acquired the SM response within six sessions. The visual aid remained in place for the remainder of the study. During in vivo instruction, because Chris only exhibited nonoccurrence of engagement, only nonoccurrence trials were initially conducted. After Chris demonstrated independent and accurate SM on nonoccurrence trials, occurrence trials were initiated by presenting a vocal prompt (i.e., “string the bead.”) for Chris to emit an occurrence of engagement. The vocal prompt was sufficient in occasioning occurrence trials, and he met the criterion for accurate SM within six sessions. When implementing the interspersed in-vivo instruction trials, the additional vocal prompt was no longer necessary because Chris independently exhibited engagement when the experimenter placed the task materials on the table. During this phase, Chris met the criterion performance within 6 sessions. SM instruction took approximately 4.25 hours for Chris. During the postinstruction SM baseline phase, Chris did not continue to self-monitor his behavior when the DRA component was removed.

Supporting Information G depicts the results of SM instruction for Bob. Bob quickly acquired the SM response when video clips depicted only nonengagement or engagement on each trial. Bob’s performance became variable when nonoccurrence and occurrence video clips were interspersed, and like Chris, Bob checked boxes on the SM worksheet out of sequence rather than sequentially. To address this, a visual aid identical to the one used with Chris was introduced for Bob. Following the introduction of this visual aid, Bob acquired the SM response during interspersed video trials. During in-vivo instruction, only interspersed trials were presented, and because Bob displayed engagement, no additional instructions were needed. Bob demonstrated independent and accurate SM on 80% of trials following eleven sessions during the in-vivo phase. SM instruction took approximately 2.58 hours for Bob. Similar to Chris, Bob did not continue to self-monitor his behavior when the DRA component was removed in the postinstruction SM baseline phase.

Supporting Information G depicts the results of SM instruction for Scott. Scott quickly acquired the SM response when video clips depicted only occurrence or nonoccurrence trials. However, his performance became more variable when the video clips were interspersed, requiring him to make a discrimination on each trial. To address the sequencing errors Scott made, a visual aid identical to that used for Chris and Bob was introduced. Following the introduction of this visual aid, Scott acquired the SM response during interspersed video trials. During in-vivo instruction with interspersed trials, Scott demonstrated independent and accurate SM on 80% of trials following nine sessions. SM instruction took approximately 3.5 hr for Scott. During the postinstruction SM baseline phase, Scott’s accurate SM maintained at moderate levels but occurred below the 80% criterion level.

Supporting Information G depicts the results of SM instruction for Josh. Josh quickly acquired the SM response when video clips depicted occurrence, nonoccurrence, and interspersed trials. During in-vivo instruction with interspersed trials, Josh demonstrated independent and accurate SM on 90% of trials following six sessions. SM instruction took approximately 3.6 hours for Josh. Josh did not self-monitor his behavior during the postinstruction SM baseline phase when the DRA component was removed.

Supporting Information G depicts the results of SM instruction for Blake. Blake acquired the SM response during video instruction sessions when the videos depicted occurrences of engagement, nonoccurrences of engagement, and interspersed trials. During in-vivo instruction with interspersed trials, Blake demonstrated independent and accurate SM on 90% of trials following four sessions. SM instruction took approximately 3.2 hours for Blake. Josh continued to self-monitor his behavior for one session during the postinstruction SM baseline phase, but then accurate SM decreased to zero.

**Supporting Information G**

*Percentage of Opportunities with Accurate Self-Monitoring During Self-Monitoring Instruction for Chris, Bob, Scott, Josh, and Blake*


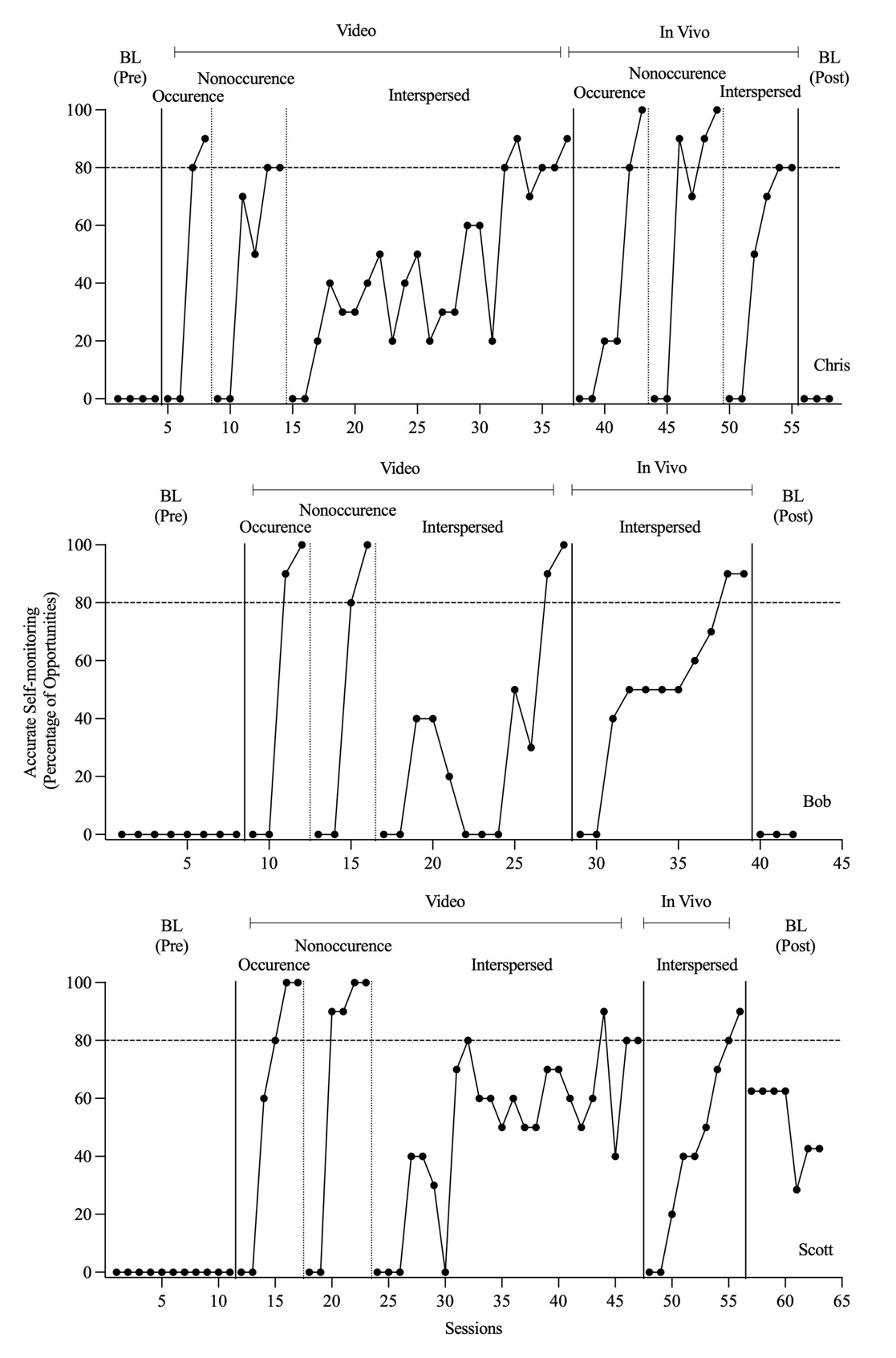

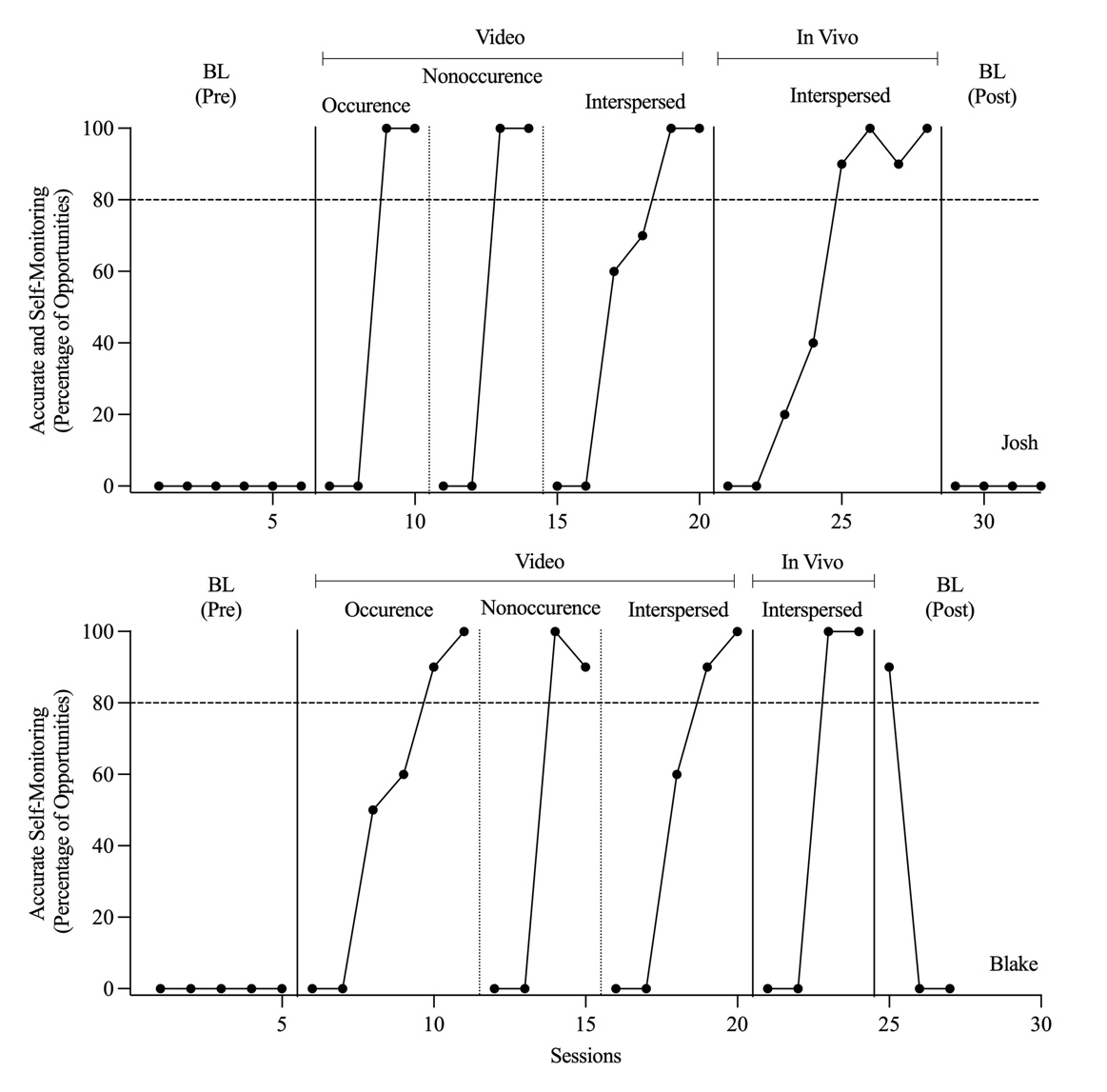


*Note.* BL = baseline; Pre = preinstruction; Post = postinstruction.

**Supporting Information H**

*Cumulative Selections During the Treatment Preference Assessment for Bob, Josh, and Blake.*

*
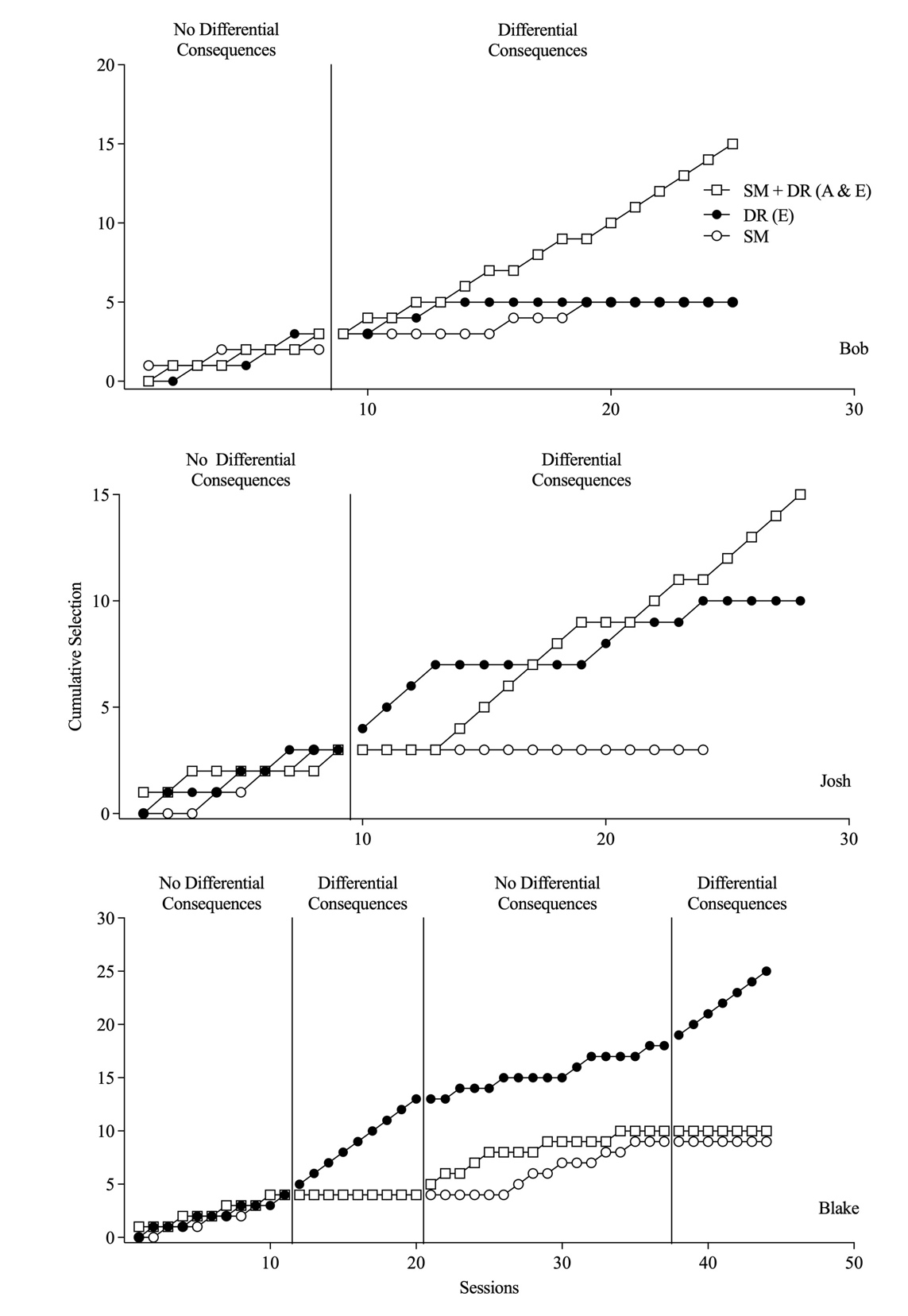
*

*Note.* SM = self-monitoring; DR = differential reinforcement of alternative behavior; A = accurate; E = engagement.
